# Genome-Wide Identification of RNA Editing Sites Affecting Muscle Development in Yak

**DOI:** 10.3389/fvets.2022.871814

**Published:** 2022-06-28

**Authors:** Xiaoyun Wu, Min Chu, Xiaoming Ma, Jie Pei, Lin Xiong, Xian Guo, Chunnian Liang, Ping Yan

**Affiliations:** Key Laboratory of Yak Breeding Engineering, Lanzhou Institute of Husbandry and Pharmaceutical Sciences, Chinese Academy of Agricultural Sciences, Lanzhou, China

**Keywords:** RNA editome, skeletal muscle, development, yak, transcriptome

## Abstract

Skeletal muscle growth and development is a complicated process that is regulated at multiple steps and by numerous myogenesis genes. RNA editing represents one of the events at the post-transcriptional level, which contributes to the diversity of transcriptome and proteome by altering the nucleotides of RNAs. However, RNA editing events in the skeletal muscle of yaks are still not well defined. This study conducted whole-genome RNA-editing identification in skeletal muscle of yaks at embryonic stage (ES) and adult stage (AS). We found a total of 11,168 unique RNA editing sites, most of which were detected in the intergenic region. After annotation, we totally identified 2,718 editing sites within coding regions, among which 858 were missense changes. Moreover, totally 322 editing sites in the 3′ untranslated regions (UTR) were also predicted to alter the set of miRNA target sites, indicating that RNA editing may be involved in translational repression or mRNA degradation. We found 838 RNA editing sites (involving 244 common genes) that are edited differentially in ES as compared to AS. According to the KEGG enrichment analysis, these differentially edited genes were mainly involved in pathways highly related to skeletal muscle development and myogenesis, including MAPK, AMPK, Wnt, and PI3K-Akt signaling pathways. Altogether, our work presents the first characterization of RNA editing sites within yak skeletal muscles on a genome-wide scale and enhances our understanding of the mechanism of skeletal muscle development and myogenesis.

## Introduction

Yak (*Bos grunniens*) is a vital species of livestock prevalent across the Qinghai-Tibet plateau and the adjacent alpine regions (1). It plays a crucial role in promoting the local economy by providing products such as meat, milk, hair, transport, and fuel for the residents. Yak meat is highly nutritious, being rich in protein and amino acids, and having low-fat content (2). Nonetheless, compared to cattle, the growth rate of yak is slow due to the lack of an efficient yak breeding program to improve the growth traits. Therefore, improving the growth rate is one of the significant breeding objectives in the yak industry.

Skeletal muscle accounts for ~40% of the bodyweight and is the main meat-producing tissue. In livestock, the development of skeletal muscle has a significant influence on the growth rate and meat yield (3). Vertebrate skeletal muscle has an essential role in locomotion and metabolism, and it originates from *paraxial mesoderm* in the embryonic stages (4). In prenatal period, increasing fiber numbers is the main characteristic of skeletal muscle growth. After birth, muscle development mainly depends on muscle fiber hypertrophy. Skeletal muscle development (myogenesis) is a complicated but orderly process involving commitment of multipotent precursor cells into myoblasts, myoblast proliferation, differentiation, fusion to myofibers, and myotubes with multiple nuclei, as well as their eventual accommodation into slow-and fast-twitch muscle fibers (5). This process is precisely orchestrated *via* several well-known transcription factors (TFs), such as the myocyte enhancer factor 2 (MEF2) family members (6), paired box protein 3/7 (Pax3/7) (7), and members of the myogenic regulatory factors (MRFs) family (8). Although many protein-encoding genes and non-coding RNAs (ncRNA) have been shown to make important contributions to the intricate process of muscle development, the distinct mechanisms have not yet been elucidated completely.

RNA editing is one of the most important post-transcriptional events altering the nucleotide composition of a transcript through the insertion, deletion, or substitution of nucleotides. It leads to differences bcetween a genomic DNA sequence and its corresponding mRNA sequence. RNA editing may occur within coding genes or non-coding regions, thereby resulting in non-synonymous substitutions, regulating alternative splicing (AS), and stability of RNAs (9). Notably, the A-to-I RNA editing is a frequently occurring RNA editing type within many animals, which is mediated by the adenosine deaminases acting on RNA (ADAR family) (10). In double-stranded RNAs (dsRNAs), ADAR family members can deaminize adenosine (A) to inosine. The latter is read by the cell machinery as guanosine (G). Additionally, cytidine-to-uridine (C-to-U) editing is also a common RNA editing type observed in animals, which is triggered by enzymes of the apolipoprotein B mRNA editing catalytic polypeptide-like (APOBEC) family (11). Recently, many bioinformatic tools and next-generation sequencing (NGS) techniques have been developed for detecting RNA editing events, which has enabled us to discover genome-wide RNA editing events and their molecular function. Presently, many studies for identification of RNA editing events have been performed in human (12), cattle (13), pigs (14), sheep (15), and chicken (16). Many studies have shown that dysregulation of RNA editing is associated with various human diseases, such as the presence of tumors (17), epilepsy (18), Alzheimer's disease (19), and sporadic amyotrophic lateral sclerosis (ALS) (20). The importance of RNA editing in myogenesis was demonstrated in a recent study by Yang et al. (21). In this study, the authors outlined the landscape of the RNA editome in porcine skeletal muscle across 27 developmental stages and identified a series of RNA editing sites related to myogenesis, indicating the crucial role of RNA editing in the process of skeletal muscle development. However, there is limited information available on the role of RNA editing in skeletal muscle development in yak. In this work, we present a genome-wide landscape of the RNA editome in yak skeletal muscle at embryonic stage (ES) and adult stage (AS) and evaluate the potential role of RNA editing events in muscle development. Our study largely extends the list of RNA editing sites in yak and provides valuable insights for better understanding of the regulatory mechanisms of muscle development in yak.

## Materials and Methods

### Data Resource

In order to investigate the RNA editing sites in muscle tissues of yak, six specific strand RNA-seq data (PRJNA550017) from our previous study (22) were downloaded from NCBI SRA (https://www.ncbi.nlm.nih.gov/sra/). This dataset was developed from *longissimus dorsi* muscle tissues of 3 embryonic (90 days old) and 3 adult (3 years old) yaks. The Illumina HiSeq 2500 platform was employed to sequence cDNA libraries of these samples (2 × 150-bp paired-end read length).

### Whole Genome Sequencing (WGS)

Blood samples were gathered from yaks having the same genetic background as the study samples used for RNA-seq. All experiments and animal care procedures were carried out according to the guidelines of the Animal Administration and Ethics Committee of Lanzhou Institute of Husbandry and Pharmaceutical Sciences of CAAS (Permit No. SYXK-2014–0002). DNA extraction kit (Tiangen Biotech, Beijing, China) was utilized to extract genomic DNA from blood in line with the specific instructions. DNA libraries were constructed with the use of the MGIEasy Universal DNA Library Prep Kit (MGI, Shenzhen, China) following the BGI's standard preparation protocol. Paired-end sequencing (2 × 150-bp) was carried out on an MGISEQ2000 (MGI, Shenzhen, China) at Frasergen Bioinformatics Co., Ltd (Wuhan, China).

### Quality Control and Reads Mapping

Reads including ploy-N, adapter or low-quality sequences were filtered out to obtain clean reads. The Burrows-Wheeler Aligner (BWA mem; version v0.7.17) (23) was used to align clean reads of WGS data to domestic yak genome sequence (LU_ Bosgru_v3.0). Samtools (Version 1.9) (24) was employed for sorting mapped reads, and duplicated reads were eliminated with Picard tools v. 2.13.2 (https://broadinstitute.github.io/picard/) using the function MarkDuplicate. We then mapped the clean reads from RNA-seq data to the genome sequence of domestic yak (LU_Bosgru_v3.0) using the HISAT2 (2.1.0) (25). The resulting SAM files were converted into BAM files using Samtools (Version 1.9) (24).

### RNA Editing Detection

In order to detect the RNA editing sites, the REDItoolDnaRna.py in REDItools v1.0.4 (26) was utilized. Parameters recommended by the researcher were chosen after mild modifications (minimal quality score (-q) 25, minimal mapping quality score (-m) 25, minimal editing frequency (-n) 0.05, minimal homopolymeric length (-O) 5, minimal read coverage (-c) 10, and minimal read number that supports variation (-v) 3) (27). Finally, only the edited sites were retained for further study, which were present in at least two samples. The SnpEff (v4.3t) (28) was employed to annotate RNA editing sites according to Ensembl-based gene annotation (release 90).

### Validation of RNA Editing Sites Through Sanger Sequencing

To validate the reliability of RNA editing sites identified in this study, three editing sites were randomly selected for PCR validation. Six independent samples used for PCR validation were collected at the same developmental stage as those used for RNA-Seq. Total RNA was extracted using the animal tissue RNA isolation kit (ZDGSY, Beijing, China) following the manufacturer's protocol. Genomic DNA was isolated using the animal tissue Genomic DNA Kit (ZDGSY, Beijing, China). Total RNA was used for reverse transcription by PrimeScript™ RT reagent kit with gDNA Eraser (TaKaRa, Dalian, China). Equal amounts of cDNA were mixed. The primer details are presented in Supplementary Table S1. The 20 μl PCR reactions contained 1 μl of mixed cDNA, 1 μl of each sense and anti-sense, 10 μl of GoTaq^®^ Green Master Mix (Promega, Madison, WI, USA) and 7 μl of ddH_2_O. The PCR program was set as follows: 95°C, 2 min, (95°C, 30s; 58°C, 30 s; 72°C, 1 min) 30 cycles and 72°C, 5 min. The PCR products were then sequenced by Sanger sequencing.

### Analyzing the Effects of RNA Editing on MiRNA Regulation

We used RNAhybrid (-b 1 -c -f 2,8 -m 100,000 -u 1 -v 1 -e−10) (29) and miRanda (sc 140 -en−10 -scale 4 -strict) (30) to detect binding targets of the miRNAs on the edited and reference sequences for editing sites in the 3'untranslated region (UTR). According to the predicted miRNA-mRNA interactions supported by both tools, the gain of miRNA binding targets was defined as the interactions existing in the edited sequences but not in the reference sequences. On the contrary, the loss of miRNA binding targets was defined as the interactions missing in the edited sequences but not in the reference sequences.

### Differential RNA-Editing Analysis

For an RNA editing site in a given sample, the RNA editing level was calculated as the ratio of the reads supporting the edited base to the total number of reads detected on this site. To identify RNA-edited sites related to muscle development, we performed Tukey's Honest Significant Difference approach to screen significantly differently edited sites between ES and AS. Sites with *P-*value < 0.05 were labeled as significant.

### Annotation and Enrichment Analysis

The genes with RNA editing sites located in exonic regions were extracted for functional enrichment analysis. To explore the functions of these putative RNA editing sites, we carried out gene ontology (GO) analysis based on the G: Profile web approach (31). KOBAS 3.0 software was employed for Kyoto Encyclopedia of Genes and Genomes (KEGG) analysis by performing a hypergeometric test (32). The cut-off value of significant GO terms and KEGG pathway was *P*-value < 0.05.

### Conservation Analysis of RNA Editing Sites

For detecting A-to-I editing events with a high conservation degree, editing sites discovered in the present work were compared with those identified in humans based on the REDIportal database (http://srv00.recas.ba.infn.it/atlas/) (33). In total, 4,627,557 editing sites in humans were adopted to conduct conservation analyses. We extracted flanking regions of 50 bp at the editing sites in domestic yak genome sequence (LU_Bosgru_v3.0) and blasted them against 50 bp flanking regions of the identified A-to I editing events in humans using the Nucleotide Basic Local Alignment Search Tool (Blastn). Hits with Expect (E) values ≤ 1e−5 and identity ≥ 85% indicated editing events with high conservation. The E-value served as a measure of the number of hits one can “expect” to see by chance when searching a database.

## Results

### RNA Editing Detection and Validation

**Figure 1:**
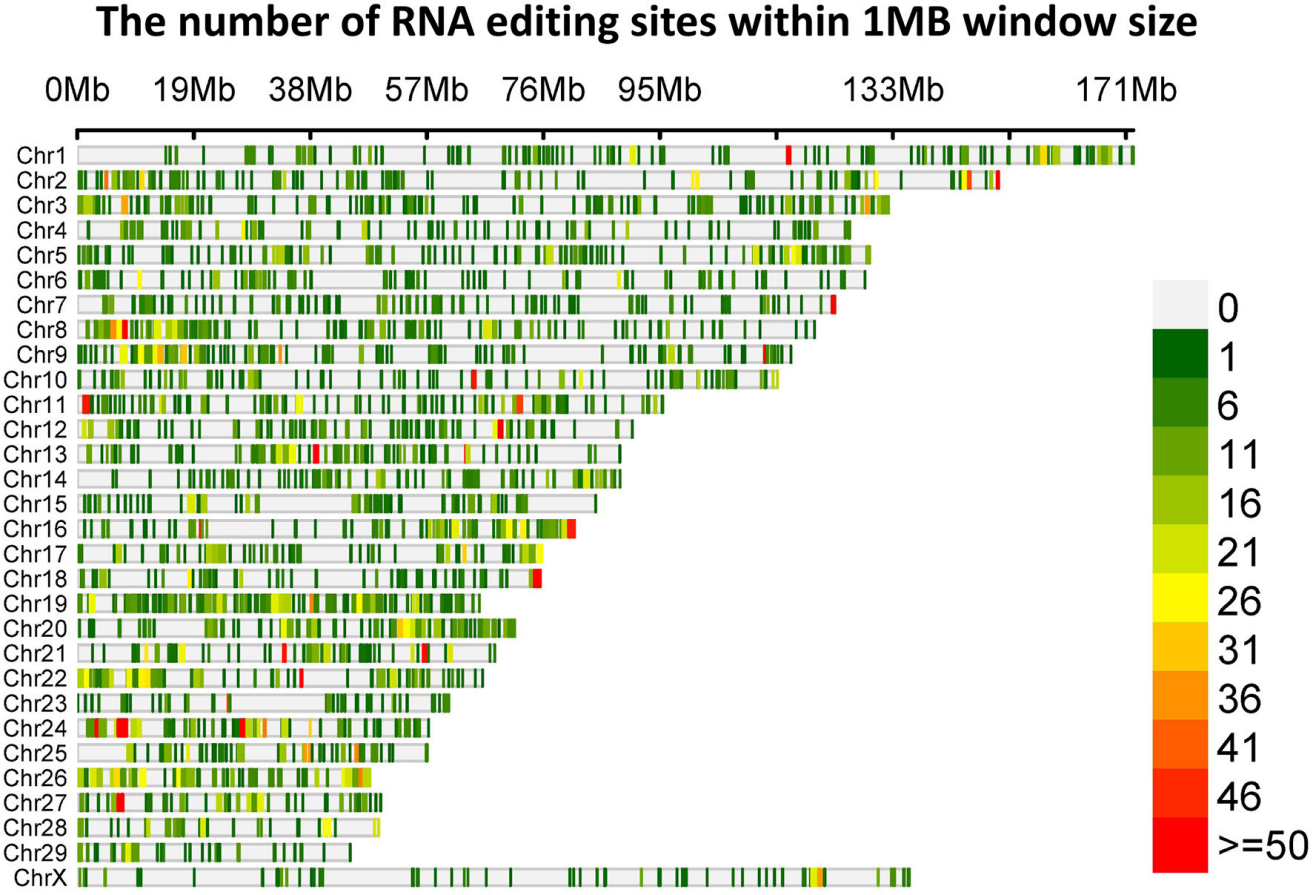
The distribution of RNA editing sites throughout the yak genomes.

**Figure 2:**
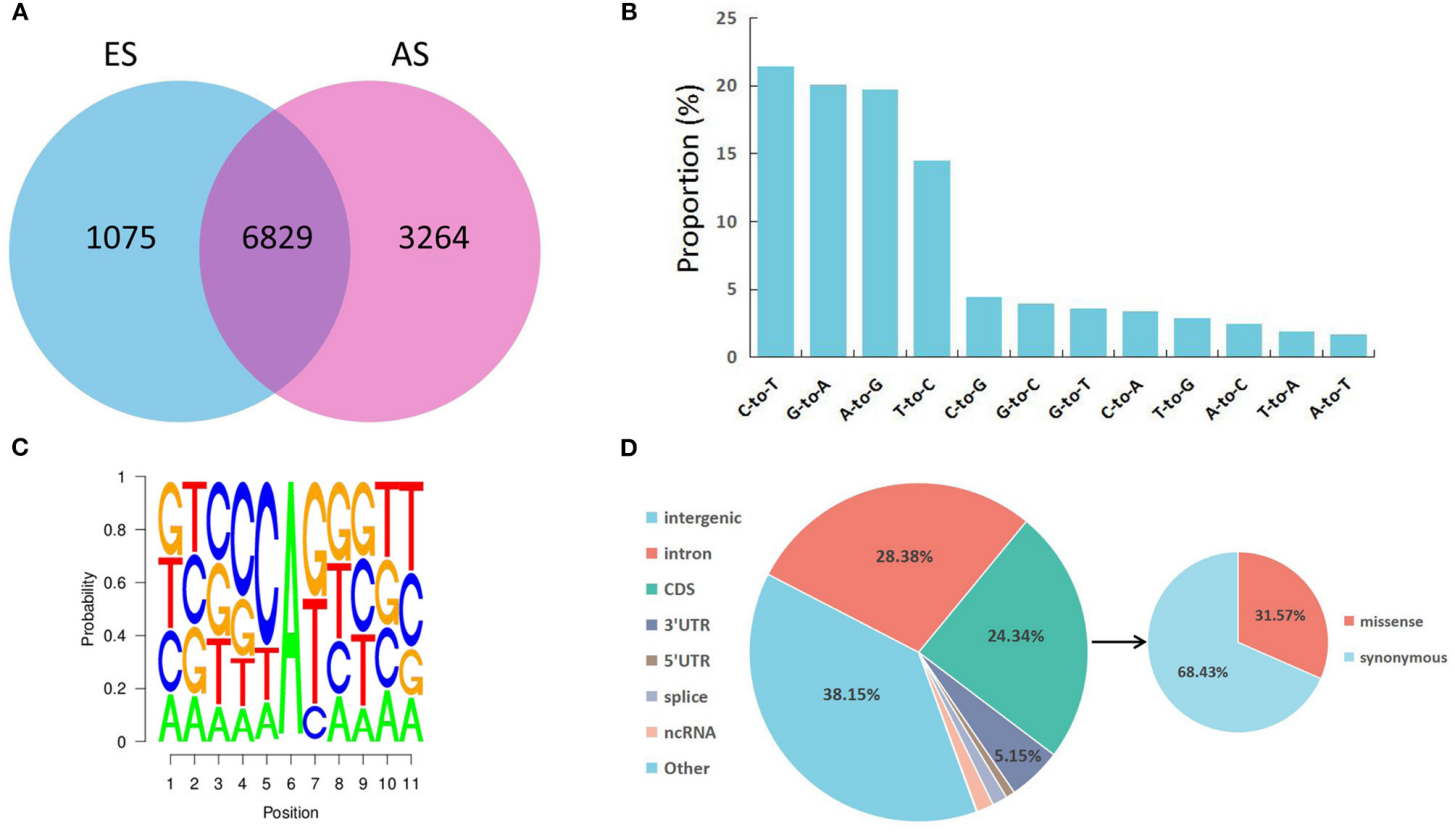
Characteristics of the editome in the skeletal muscle. **(A)** Venn diagram showing the number of shared editing sites between AS and ES; **(B)** Proportion of 12 types of RNA editing; **(C)** Neighbor sequence preferences of A-to-G RNA editing; **(D)** Distribution of the identified RNA editing sites throughout the genome.

To determine RNA editing sites at the genomic level in the *longissimus dorsi* muscle, we collected six strand-specific RNA sequencing samples from both the ES and AS. After trimming the adaptor sequence and low-quality reads, we obtained 282,913,382 total clean reads for ES and 363,479,946 total clean reads for AS. The total mapped ratio between the reads and the reference genome of all samples ranged from 88.19 to 92.58% (Table 1). We identified a total of 31,244 raw editing sites using strict filtering criteria (Supplementary Table S2). After potential SNPs filtering, 11,168 high-confidence RNA editing events were kept (Supplementary Table S3). The ES1 individual showed the fewest, whereas the AS1 individual showed the highest number of editing events. Additionally, these RNA editing events showed a non-uniform location within the yak chromosomes (Figure 1). The highest number of editing sites (670) was observed on chromosome 8, while the least number of editing sites (126) was found on chromosome 29. As our expectation, the number of RNA editing sites was different between ES and AS. Our analysis indicates that 6,829 RNA editing sites were shared between the two groups, 1,075 editing sites were specific to ES, and 3,264 editing sites were specific to AS (Figure 2A). A total of 12 different types of RNA editing were identified, each with a proportion >1%. Over 40% of these sites correspond to A-to-G and C-to-T type, consistent with A-to-I and C-to-U editing (Figure 2B). To validate the predicted RNA editing sites, three sites were randomly selected for PCR and Sanger sequencing. The sites were considered to be successfully verified if the cDNA sequence was heterozygous while the corresponding DNA sequencing was homozygous. After aligning the cDNA and DNA sequences, selected editing sites were consistent with the prediction (Supplementary Figure S1).

**Table 1:** Summary of RNA-Seq data and mapping.

**Sample name**	**Clean reads**	**Total mapped**	**Uniquely mapped**
AS1	122,761,590	88.59%	69.96%
AS2	121,063,908	88.19%	70.11%
AS3	119,654,448	88.50%	70.71%
ES1	94,636,614	92.58%	70.82%
ES2	94,667,640	91.40%	72.67%
ES3	93,609,128	92.32%	72.03%

### Sequence Preference and Annotation of RNA Editing Sites in Skeletal Muscle

As shown in Figure 2C, G is underrepresented at −1 position and overrepresented at +1 position of the A-to-G editing sites discovered in our study. The identified sequence preference for A-to-G editing sites corresponds to the findings of previous reports on mammalian ADAR (34, 35). To characterize the distribution of RNA editing sites, the SnpEff tool was applied to identify each RNA editing site that corresponded to the annotated gene. Totally RNA editing sites overlapped with 2,584 annotated genes. The RNA editing sites identified in this study were annotated to 7 types of genomic locations: intergenic regions, introns, coding sequences (CDS), 3′ UTRs, 5′ UTRs, ncRNA, and splice sites (Supplementary Table S4). Most RNA editing sites were localized in the intergenic region (38.15%), followed by introns (28.38%) (Figure 2D). Furthermore, a total of 858 editing events residing in CDS were identified as missense editing events, which could influence the functions of protein.

### Cross-Species Analysis Between Yak and Human

To investigate the conservation of RNA editomes between human and yak, upstream and downstream sequence of editing sites was aligned using nucleotide BLAST tools. With a rigorous filtering threshold (identity >85%, *E*-value <1e−5), totally eight conserved RNA editing sites were identified (Supplementary Table S5). Of these, editing site in the COPI coat complex subunit alpha (*COPA*) gene resulted in missense substitution.

### Impact of RNA Editing on MiRNA Regulation

**Figure 3:**
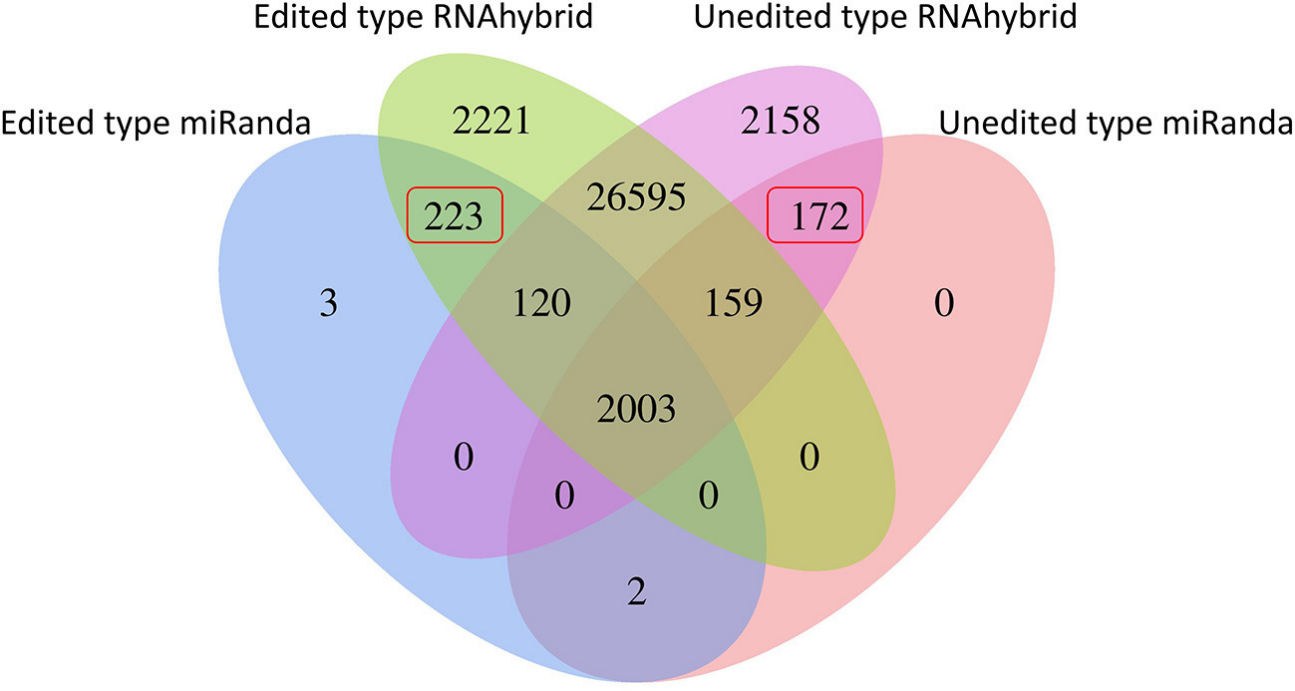
Statistical features of the RNA editing sites that changed miRNA binding capacity.

RNA editing events residing in miRNA binding sites could change the miRNA-mRNA interactions and modulate translational suppression or mRNA degradation. For the RNA editing sites residing in 3' UTRs, TargetScan and miRanda were adopted for detecting the miRNA binding targets for each reference sequence. By predicting miRNA targets, 232 editing sites were presumed to generate 223 novel miRNA binding sites and abolish 172 primitive ones (Figure 3; Supplementary Table S6). For a better understanding of the functional roles of these 190 target genes, GO and KEGG pathway enrichment analyses were performed. As shown in Supplementary Figure S2, these target genes were categorized into two categories: biological process (BP) and cellular component (CC). The five most significantly enriched terms in the BP category were macromolecule localization, cellular macromolecule localization, cellular localization, localization, and protein localization. According to KEGG analyses, the impacted target genes were mostly enriched in pathways associated with muscle development, such as mTOR, Notch, and insulin signaling pathways (Supplementary Figure S3).

### Differential RNA-Editing Analysis in Skeletal Muscle at Different Periods

**Figure 4:**
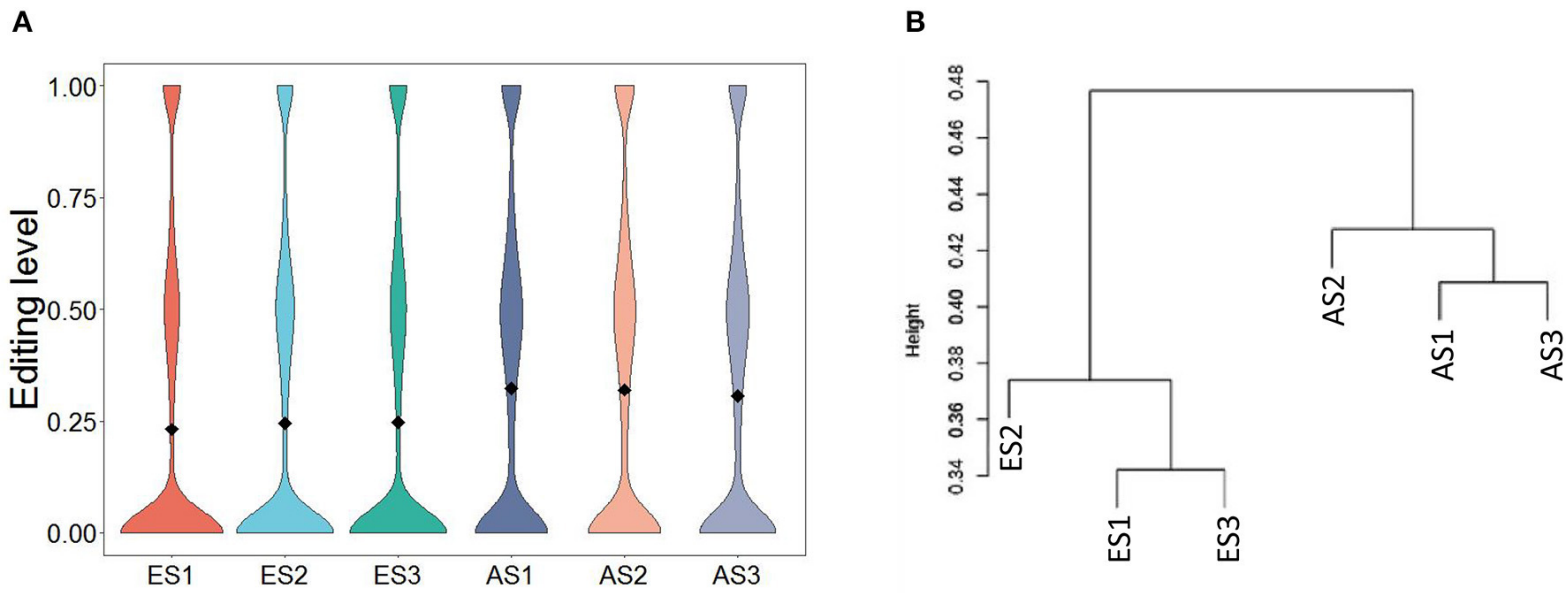
Characteristics of RNA editing levels within and between groups. **(A)** Distribution of editing levels across samples; **(B)** Hierarchical clustering of RNA editing levels across samples.

**Figure 5:**
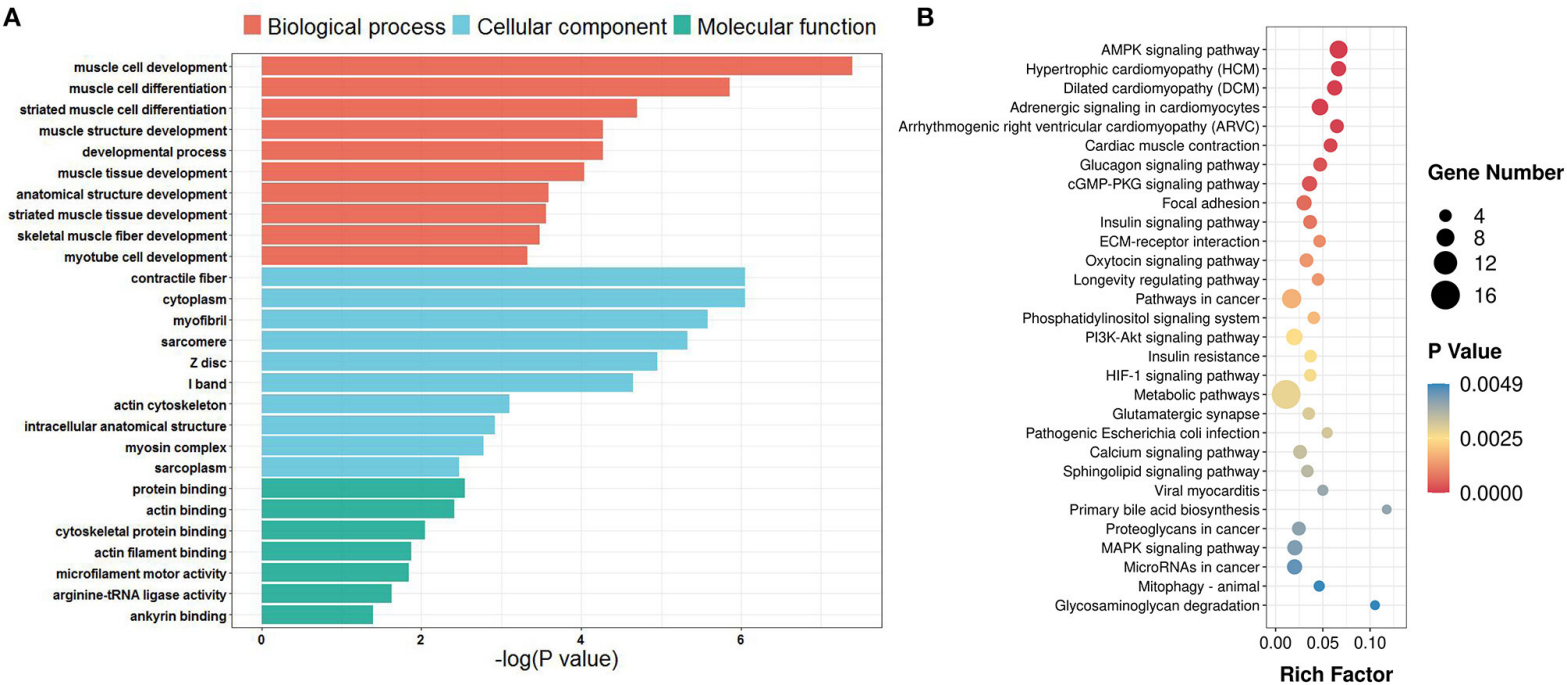
GO and KEGG enrichment of genes with differential editing levels. **(A)** GO enrichment terms; **(B)** KEGG pathway enrichment terms. Rich Factor is the ratio of differentially edited gene numbers annotated in this pathway term to all gene numbers annotated in this pathway term.

The average editing level for each sample ranged from 0.23 to 0.32 (Figure 4A). Based on hierarchical clustering, it could be found that the discrepancies in the editing levels within groups were less than those between groups (Figure 4B). These results suggest that RNA editing sites at the genome-wide level can be adopted for characterizing the developmental stage of skeletal muscle. To identify the muscle development-associated RNA editing events, this work utilized Tukey's Honest Significant Difference approach to search for the differential editing sites between ES and AS. We discovered a total of 838 differential editing events (Supplementary Table S7). GO analysis indicated that these genes with differential editing levels were involved in 120 terms (Supplementary Table S8). In the BP category, muscle cell development, muscle cell differentiation, and striated muscle cell differentiation were the most abundant terms (Figure 5A). KEGG pathway analyses demonstrated that these differentially edited genes were significantly enriched in muscle development-related pathways, such as AMPK signaling pathway, focal adhesion, MAPK signaling pathway, insulin signaling pathways, ECM-receptor interaction, Wnt signaling pathway and PI3K-Akt signaling pathway (Figure 5B; Supplementary Table S9).

## Discussion

RNA editing represents one of the important modifications at the post-transcriptional level, and it alters RNA nucleotide sequences, thereby impacting the mRNA structure and proteomic diversity. It was first identified in trypanosomes (36). Thanks to the continuous advancements in NGS technologies, many RNA editing events have been discovered in animals (37) and plants (38). To date, however, RNA editing in yak has been far less studied. To explore how RNA editing affects the muscle development processes in yak, we identified RNA editing sites at ES and AS at the whole-sequence level.

The present study discovered 11,168 editing events in yak *longissimus dorsi* muscle. It is worth noting that there are lesser editing sites at embryonic stage as compared with the adult stage, which suggests the decreased activity of RNA editing at embryonic stage. Notably, the two canonical RNA editing events, viz., A-to-I and C-to-U, occupy up to 41.12% of those putative editing events, indicating that the accuracy of our results is high. Additionally, eight RNA editing sites were conserved between yak and human. Consistent with the results obtained for previous studies (13, 21), the low level of overlap between editing sites identified in this study and the reported human editing sites suggests that most RNA editing sites are poorly conserved through evolution.

Unlike other studies in which the intronic region includes the most RNA editing sites (39), our study indicated that the intergenic region (38.15%) had the most RNA editing sites, followed by the intronic region (28.38%). Two possible explanations may account for this discrepancy. The first is that the annotation of the yak reference genome was inaccurate and incomplete compared with the human reference genome. The second possibility is that our RNA-seq libraries which were prepared with RiboZero rRNA Removal Kit, contain a large number of intergenic lncRNAs. However, our results indicate a potentially relationship between RNA editing events that reside in non-coding regions and the regulation of gene expression.

The recoding RNA editing sites lead to non-synonymous replacements that increase proteomic diversity (40). It is reported that numerous recoding RNA editing sites are preserved and have functional and evolutionary importance (41, 42). Altogether 858 recoded RNA editing sites were identified within 650 genes, some of which were related to skeletal muscle development. For example, myosin heavy chain 3 (*MYH3*) belongs to the myosin heavy chain (MYH) family and is predominantly expressed in distinct muscle developmental stages. Missense mutations in the *MYH3* gene have been reported to give rise to human muscle development disorders (43). Sad1 And UNC84 Domain Containing 1 (*SUN1*) belongs to the linker of nucleoskeleton and cytoskeleton (LINC) complex, which plays a critical role in myotube formation (44). A previous study revealed that loss of SUN1 leads to myofibers with a smaller diameter, and this slows down the adult skeletal muscle regeneration (45). Myosin 18B (*MYO18B*) is the new nontraditional myosin heavy chain that is mostly expressed in human cardiac and skeletal muscle (46). Disruption of *MYO18B* could suppress the proliferation and differentiation of C2C12 mouse myoblasts (47). Therefore, these recoding RNA editing sites may have a crucial role in the skeletal muscle development in the yak. Except for these recoding editing sites, some RNA editing sites within the 3′ UTR of mRNAs could regulate the expression of target genes by creating or destroying miRNA binding sites (40). In the present study, we discovered 253 RNA editing sites that might alter target gene expression by changing the power of miRNA binding. The KEGG enrichment analysis indicated that the impacted target genes were enriched in some critical pathways associated with muscle development, including hippo (48), mTOR, and insulin signaling pathways (49). These results suggest that RNA editing events within miRNA binding sites might regulate skeletal muscle development *via* translational repression or mRNA degradation.

In this study, a total of 838 sites were found to be differentially edited between the AS and ES groups. According to KEGG analyses, genes that showed different editing levels were mostly enriched in several important pathways associated with muscle development, e.g., MAPK, AMPK, Wnt and PI3K-Akt signaling pathways and ECM-receptor interaction. The MAPK signaling pathway was considered to be a crucial regulator of skeletal muscle growth and development (50). Myocyte enhancer factor 2C (MEF2C) and voltage-dependent, alpha-2/delta subunit 1(CACNA2D1) are involves in MAPK signaling pathway. MEF2C belongs to the MADS-box transcription enhancer factor 2 (MEF2) family, which is known to be involved in myogenesis. It was found that miR-204–5p can inhabit the myoblast differentiation through repression *MEF2C* gene (51). CACNA2D1 belongs to the alpha-2/delta subunit family, which is associated with voltage-gated calcium channels. Variations of the *CACNA2D1* gene are significantly associated with bovine carcass traits (52). Laminin 2 (*LAMA2*) encodes an important extracellular matrix (ECM) protein which is mainly expressed in the basement membrane of skeletal muscle (53). Variants in *LAMA2* were identified to be the causal mutations of congenital merosin-deficient muscular dystrophy (54). These observations suggest that RNA editing may influence skeletal muscle development and myogenesis.

## Conclusion

In conclusion, the present study comprehensively analyzes the RNA editome in the skeletal muscle of yaks at ES and AS. We identified 11,168 high-confidence RNA editing sites. Of these, many RNA editing sites with different editing levels may potentially contribute to myogenesis and muscle development. However, their biological functions and regulatory mechanisms need to be further investigated in depth. Our research expands the list of RNA editing sites in yak and offers profounder insight into understanding the mechanism of yak muscle development.

## Data Availability Statement

The original contributions presented in the study are included in the article/Supplementary Material, further inquiries can be directed to the corresponding authors.

## Ethics Statement

The animal study was reviewed and approved by Animal Administration and Ethics Committee of Lanzhou Institute of Husbandry and Pharmaceutical Sciences of CAAS (Permit No. SYXK-2014–0002).

## Author Contributions

XW, PY, and CL conceived the research and drafted the manuscript with comments from all authors. XW, MC, and XM conducted the experiments and analyses. JP, LX, and XG participated in experiments and revised the manuscript. All authors approved the final version.

## Funding

This study was supported by the National Natural Science Foundation of China (32102500), Agricultural Science and Technology Innovation Program (25-LZIHPS-01), Gansu Province Science Foundation for Youths (20JR5RA576), Foundation for Innovation, Groups of Basic Research in Gansu Province (20JR5RA580), and Central Public-interest Scientific Institution Basal Research Fund (Y2022LM10).

## Conflict of Interest

The authors declare that the research was conducted in the absence of any commercial or financial relationships that could be construed as a potential conflict of interest.

## Publisher's Note

All claims expressed in this article are solely those of the authors and do not necessarily represent those of their affiliated organizations, or those of the publisher, the editors and the reviewers. Any product that may be evaluated in this article, or claim that may be made by its manufacturer, is not guaranteed or endorsed by the publisher.
