# Supplementary material for: Genome-Wide Identification of RNA Editing Sites Affecting Muscle Development in Yak
Source: Front Vet Sci. 2022 Jun 28;9:871814. doi: 10.3389/fvets.2022.871814 (PMC9274240; doi:10.3389/fvets.2022.871814)
Supplement: Supplementary file 1 [file Data_Sheet_1.PDF]

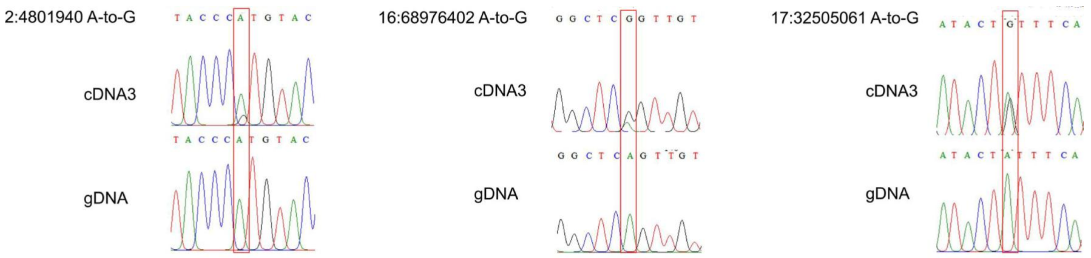

FIGURE S1 | Validation of RNA editing sites by PCR and Sanger sequencing

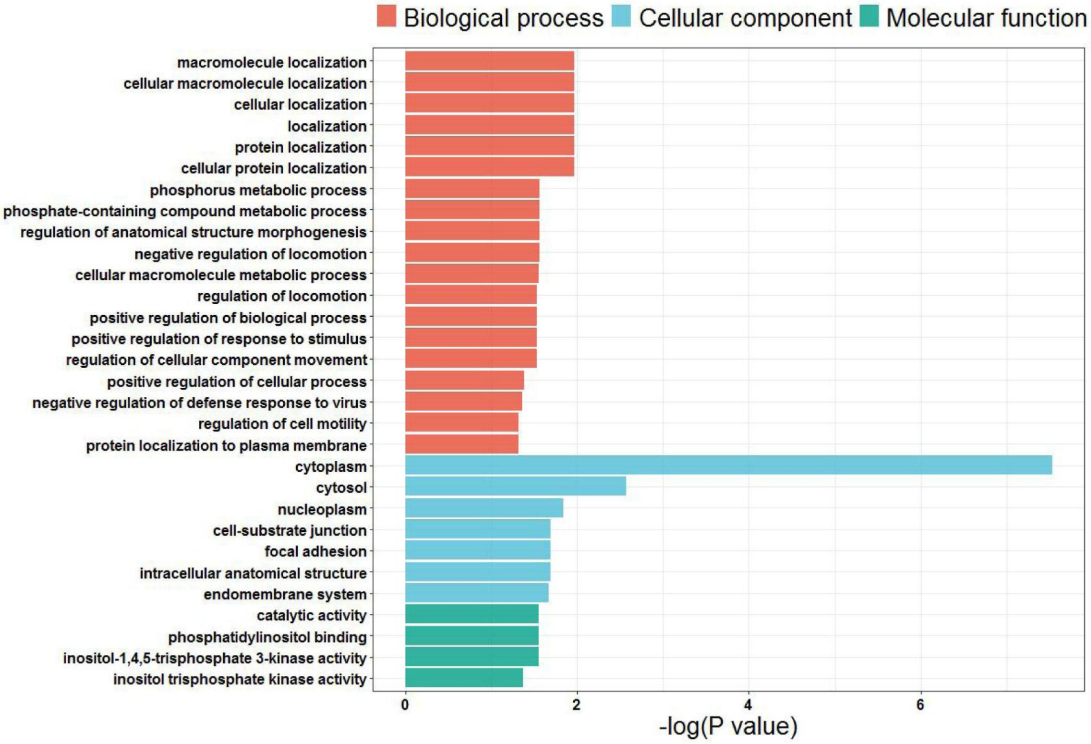

FIGURE S2 | GO enrichment of genes with modified miRNA binding sites.

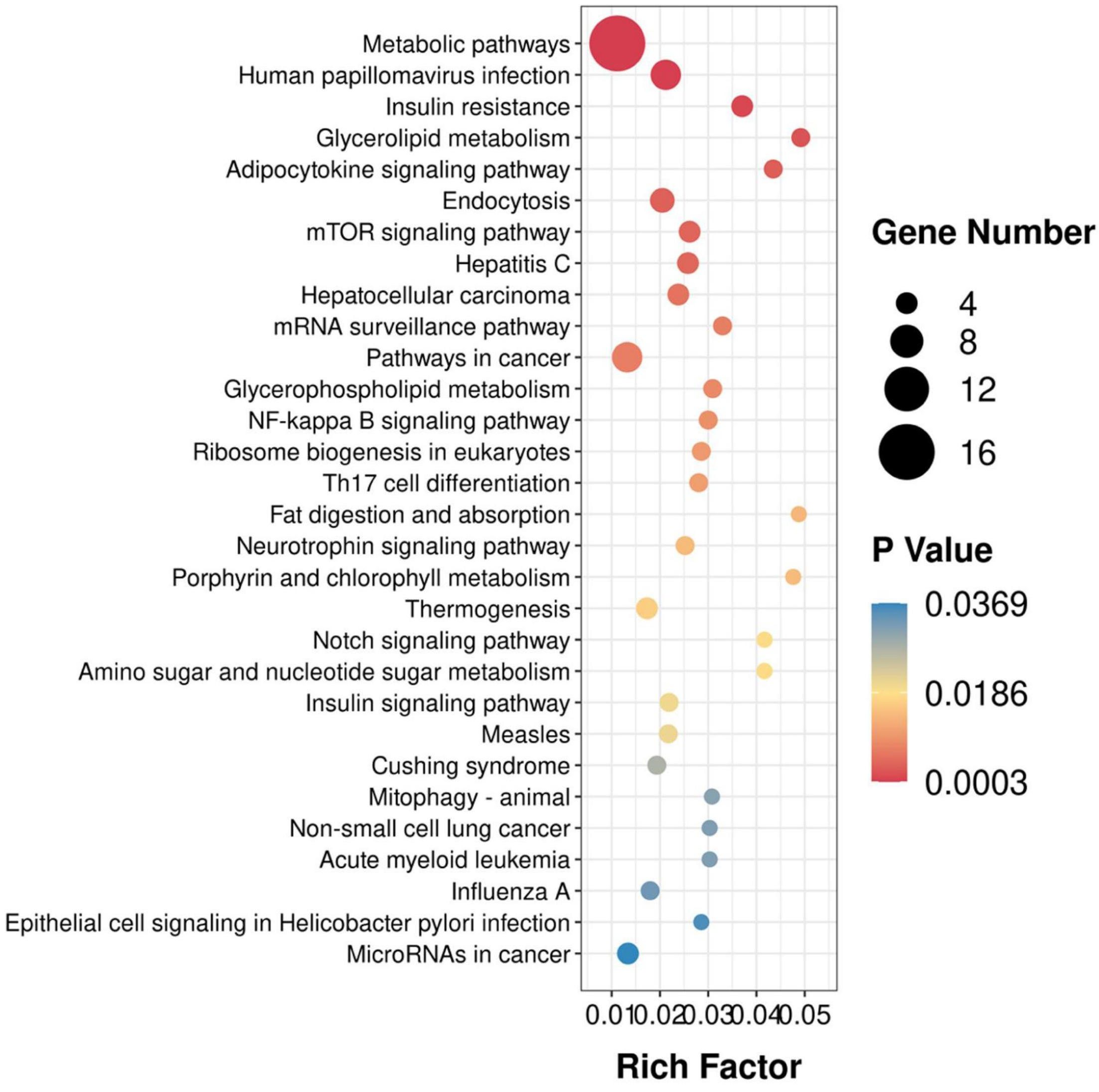

FIGURE S3 | KEGG enrichment of genes with modified miRNA binding sites.
